# Smoldering Multiple Myeloma

**DOI:** 10.1155/2015/623254

**Published:** 2015-04-27

**Authors:** Minjie Gao, Guang Yang, Yuanyuan Kong, Xiaosong Wu, Jumei Shi

**Affiliations:** Department of Hematology, Shanghai Tenth People's Hospital, Tongji University School of Medicine, Shanghai 200072, China

## Abstract

Smoldering multiple myeloma (SMM) is an asymptomatic precursor stage of multiple myeloma (MM) characterized by clonal bone marrow plasma cells (BMPC) ≥ 10% and/or M protein level ≥ 30 g/L in the absence of end organ damage. It represents an intermediate stage between monoclonal gammopathy of undetermined significance (MGUS) and symptomatic MM. The risk of progression to symptomatic MM is not uniform, and several parameters have been reported to predict the risk of progression. These include the level of M protein and the percentage of BMPC, the proportion of immunophenotypically aberrant plasma cells, and the presence of immunoparesis, free light-chain (FLC) ratio, peripheral blood plasma cells (PBPC), pattern of serum M protein evolution, abnormal magnetic resonance imaging (MRI), cytogenetic abnormalities, IgA isotype, and Bence Jones proteinuria. So far treatment is still not recommended for SMM, because several trials suggested that patients with SMM do not benefit from early treatment. However, the Mateos et al. trial showed a survival benefit after early treatment with lenalidomide plus dexamethasone in patients with high-risk SMM. This trial has prompted a reevaluation of early treatment in an asymptomatic patient population.

## 1. Introduction

Smoldering multiple myeloma (SMM) was first described in 1980 in the New England Journal of Medicine (NEJM) [1]. Its definition varied, but the universal recognition was that SMM exceeded the limits of the definition of monoclonal gammopathy of undetermined significance (MGUS) and had no end-organ damage. In 2003 the International Myeloma Working Group (IMWG) provided the criteria that SMM was defined as clonal bone marrow plasma cells (BMPC) ≥ 10% and/or M protein level ≥ 30 g/L and lack of end organ damage (CRAB—hypercalcemia, renal failure, anemia, and bone lesions) [2]. SMM accounts for about 15% of all the patients with newly diagnosed MM [3]. The risk of progression to symptomatic MM is markedly higher in SMM compared to MGUS, 10% per year versus 1% per year, respectively [4]. Currently, patients with SMM are not treated until the development of MM symptoms. In the past, some trials used alkylating agents such as melphalan to evaluate the effect of early treatment on patients with SMM [5–7]. They caused obvious toxicity and failed to show a significant benefit. With the introduction of novel agents, investigators attempted early treatment with novel agents such as thalidomide for patients with SMM, but early treatment still did not result in improved survival benefit [8–11]. However, Mateos et al. reported the results of their phase III trial and showed that early treatment for patients with high-risk SMM improved overall survival [12]. This prompted a reconsideration of treatment of SMM and the definition the high-risk SMM. This purpose of this review is to summarize and evaluate the prognostic factors predicting progression to active MM, to discuss early treatment of patients with SMM, and to provide directions for further investigations.

## 2. Definition of SMM

In 1980, Kyle and Greipp first introduce the concept of smoldering (asymptomatic) multiple myeloma (SMM) in defining six myeloma patients in whom the percentage of plasma cells and level of M protein were higher than those seen in MGUS and fulfilled the criteria for diagnosis of MM but have no anemia, hypercalcemia, and lytic bone lesions. These patients remained stable without specific therapy for five or more years [1]. At the same time, Alexanian et al. use the term indolent multiple myeloma (IMM) in defining 20 patients who were asymptomatic from their low tumor mass disease, had a hemoglobin greater than 10 g/dL, and showed no recurrent infection, painful compression fractures, or more than 3 lytic bone lesions [13]. In 1988, Alexanian et al. made changes to the definition of SMM and IMM, distinguishing SMM from IMM [14]. Before 2003, many studies used different criteria to define asymptomatic patients with myeloma. In 2003 IMWG defined SMM as BMPC ≥ 10% and/or M protein level ≥ 30 g/L and lack of end organ damage (CRAB—hypercalcemia, renal failure, anemia, and bone lesions) [2].

## 3. Predictors of Progression to Active MM

Most of the patients diagnosed with SMM will progress to symptomatic MM. However, SMM is not a uniform disease and patients with SMM do not progress to symptomatic MM at the same rate. Hence, it is important to define the risk of progression. A number of parameters have been described to predict risk of progression to symptomatic MM (Table 1).

### 3.1. The Level of Serum M Protein and the Percentage of Bone Marrow Plasma Cells

The level of serum M protein and the percentage of BMPC were associated with the risk of progression from SMM to active disease. Kyle et al. divided patients with SMM into three prognostic groups by the percentage of BMPC and level of serum M protein (group 1: BMPC ≥ 10% and serum M protein ≥ 3 g/dL; group 2: BMPC ≥ 10% but serum M protein < 3 g/dL; group 3: serum M protein ≥ 3 g/dL but BMPC < 10%). The median time to progression (TTP) in groups 1, 2, and 3 were 2, 8, and 19 years, respectively [15]. Kastritis et al. reported the median TTP for patients with BMPC ≥ 10% and serum M protein ≥ 3 g/dL was 19 months versus 73 months for patients with BMPC ≥ 10% but serum M protein < 3 g/dL. They identified that 8% of patients had BMPC ≥ 60% at diagnosis of SMM and these patients had a median TTP of 15 months [16]. Rajkumar et al. studied 655 patients with SMM and found that the median TTP to active myeloma was significantly shorter for patients with BMPC ≥ 60%, as compared with those with BMPC < 60% (*P* < 0.001) [17]. It was suggested that SMM should be defined with an upper limit of BMPC and patients with BMPC ≥ 60% should not be considered as asymptomatic MM and should receive therapy immediately [16–18].

### 3.2. Immunophenotyping and Immunoparesis

The proportion of immunophenotypically aberrant plasma cells (defined as elevated expression of CD56, the absence of CD45 and/or CD19, and decreased expression of CD38) within the bone marrow plasma cells (aPCs/BMPC) ≥ 95% and immunoparesis (reduction of one or more uninvolved immunoglobulin isotypes below the lower limit of normal) were risk factors for progression. Pèrez-Persona et al. reported that a risk of progression to active MM at 5 years of 4%, 46%, and 72%, respectively, for patients with none, 1, or 2 risk factors (aPCs/BMPC ≥ 95% and immunoparesis) [19]. Over time, Pèrez-Persona et al. reported that in the evolving SMM arm (defined as an increase in the level of serum M protein of at least 10% during the first 6 months of follow up, or a progressive and constant increase of the M component until overt MM developed), the 3-year progression rate for patients with aPCs/BMPC ≥ 95% was 46% versus 8% for those patients with aPCs/BMPC < 95% (*P* = 0.01). This was also observed in the nonevolving SMM arm, in which the 3-year progression rate was 15% for patients with aPCs/BMPC ≥ 95%, whereas no progression was observed in the aPCs/BMPC < 95% arm (*P* = 0.01) [20].

### 3.3. Serum-Free Light-Chain Ratio

The serum immunoglobulin FLC ratio was an independent predictor of progression to active MM. Larsen et al. concluded that a high serum-free light-chain (FLC) ratio ≥ 100 was a biomarker of early progression to active MM. The median TTP in the FLC ratio ≥ 100 arm was 15 months versus 55 months in the FLC < 100 arm (*P* < 0.0001) [21]. Kastritis et al. reported that abnormal FLC ratio was one of the most significant factors for progression, reporting that the median TTP in the FLC ratio ≥ 8 arm was 55 months versus 73 months in the FLC ratio < 8 arm (*P* < 0.005). The median TTP for patients with a FLC ratio ≥ 100 was 18 versus 73 months for patients with FLC ratio < 100 [16]. Dispenzieri et al. constructed a risk-stratification model based on the risk factors (BMPC ≥ 10%; serum M protein ≥ 3 g/dL; and FLC ratio > 8). The 5-year progression rates for the presence of one, two, or three risk factors were 25%, 51%, and 76%, respectively [22].

### 3.4. Circulating Peripheral Blood Plasma Cells (PBPC)

Detection of circulating peripheral blood plasma cells (PBPC) helped to predict disease course in patients with SMM. Witzig et al. detected the number of PBPC in 57 patients with SMM and found that the median TTP for patients with abnormal PBPC (defined as an increase in number or proliferative rate of PBPC) was 0.75 years versus 2.5 years for those patients without abnormal PBPC (*P* < 0.01) [23]. Bianchi et al. concluded that high PBPC (defined as absolute PBPC > 5000 × 10^6^/L and/or >5% cytoplasmic immunoglobulin (Ig) positive plasma cells per 100 peripheral blood mononuclear cells) could predict the evolution of SMM to active MM. The progression rate at two years for patients with high PBPC was 71% versus 25% without high PBPC (*P* = 0.001). Corresponding progression rate at 3 years were 86% versus 35%, respectively (*P* < 0.001) [24].

### 3.5. Pattern of Serum M Protein Evolution

Based on the pattern of changes in serum M protein, SMM was classified into evolving SMM and nonevolving SMM. Evolving SMM was characterized by a progressive increase in serum M protein, a previously recognized MGUS, and a significantly higher proportion of IgA type. Nonevolving SMM was characterized by stable M protein until disease progression. The median TTP for patients with evolving SMM was 1.3 years versus 3.9 years for those with nonevolving SMM (*P* = 0.007) [25].

### 3.6. Novel Imaging Assessments

Moulopoulos et al. assessed the prognostic significance of magnetic resonance imaging (MRI) in patients with asymptomatic myeloma and reported that the median TTP for patients with abnormal MRI was 16 months versus 43 months for those with normal MRI (*P* < 0.01) [26]. Hillengass et al. analyzed the prognostic significance of focal lesions in whole-body MRI in patients with SMM and founded that the detection of more than one focal lesions was the adverse prognostic factors for progression into SMM (*P* < 0.001). The 2-year progression rate for patients with 0 or 1 focal lesion was 20% versus 70% for those patients with >1 focal lesion [27]. Kastritis et al. reported that abnormal marrow signal of MRI of the spine was associated with a significant factor for progression to symptomatic myeloma (median 15 months, *P* = 0.001) [16].

### 3.7. Cytogenetic Abnormalities

Neben et al. analyzed the impact of chromosomal aberrations on progression in patients with SMM and found that the presence of del (17p13), t(4;14), +1q21 and hyperdiploidy predicted shorter TTP. The median TTP for patients with del (17p13) was 2.04 years (versus 5.62 years without del (17p13), *P* = 0.001), and with t(4;14) was 2.91 years (versus 5.71 years without t(4;14), *P* = 0.003). The progression rate at three years for patients with +1q21 was 43% (versus 27% without +1q21, *P* = 0.02), and 35% for those with hyperdiploidy (versus 29% without hyperdiploidy, *P* = 0.016) [28]. Rajkumar et al. reported that the median TTP to SMM was 28 months with t(4;14), 34 months with trisomies alone, 55 months with t(11;14), MAF translocations, other/unknown IgH translocations, monosomy 13/del (13q) without other abnormalities, and those with both trisomies and IgH translocations, but a median TTP was not reached in patients without detectable abnormalities, *P* = 0.001. There was a trend to shorter TTP with deletion 17p (median TTP, 24 months) [29].

### 3.8. IgA Isotype

Whether IgA isotype was a harmful prognostic factor for progression of SMM to active MM was unclear. Weber et al. assessed the clinical features in 101 patients with SMM and found that IgA isotype was present in 23 of 101 patients. There was a trend that the median TTP for patients with IgA isotype was shorter than those with IgG isotype (21 months versus 36 months, *P* = 0.21) [30].

### 3.9. Bence Jones Proteinuria

The predictive role for Bence Jones proteinuria was controversial and of uncertain importance. Dimopoulos et al. reported that Bence Jones proteinuria represented an independent prognostic factor. Bence Jones protein > 50 mg/d was present in 28 of 95 patients. The median TTP for patients with Bence Jones protein > 50 mg/d was 19 months versus 29 months for those with Bence Jones protein ≤ 50 mg/d (*P* = 0.02) [31].

## 4. Results of Interventional Therapeutic Trials

SMM has no obvious symptoms and may require different treatment strategies. For patients with SMM, it is not clear whether it is better to immediately treat after diagnosis or to delay treatment until symptomatic MM develops. Many investigators performed clinical trials to determine whether early treatment using conventional and novel agents resulted in improved clinical outcomes for patients with SMM, when compared with deferred treatment (Table 2).

### 4.1. Melphalan and Prednisone

Three small randomized studies [5–7] compared early treatment with melphalan and prednisone with deferred therapy until disease progression. All three trials showed no significant improvement in TTP or overall survival (OS).

### 4.2. Bisphosphonates

Several trials compared single agent bisphosphonate with observation in patients with SMM. Martin et al. performed a single-arm pilot study to assess the effects of pamidronate on 12 patients with SMM or IMM and suggested that pamidronate treatment reduced bone turnover but had no significant antitumour effect [32]. Two random trials also compared pamidronate with observation in patients with SMM and founded that pamidronate decreases the development of skeletal events but did not delay disease progression and improve OS [33, 34]. Musto et al. evaluated zoledronic acid versus observation in patients with SMM and founded that zoledronic acid reduced the rate of skeletal-related events but did not influence the natural history of SMM [35].

### 4.3. Thalidomide

Three nonrandomized, phase II trials evaluating thalidomide-based treatment suggested that long-term thalidomide therapy was poorly tolerated and most of the patients discontinued treatment due to peripheral neuropathy [8–10]. A randomized study compared combination therapy consisting of thalidomide and zoledronic acid with zoledronic acid alone in patients with SMM. The overall response rate after one year in the thalidomide plus zoledronic acid arm was 37% versus 0% in the zoledronic acid alone arm (*P* = 0.0004). The median TTP was significantly longer in the thalidomide plus zoledronic acid arm than in the zoledronic acid alone arm (2.4 versus 1.2 years, *P* = 0.02). The one-year progression-free survival (PFS) in the thalidomide plus zoledronic acid arm was 86% versus 55% in the zoledronic acid alone arm (*P* = 0.0048). There was no difference in OS between the arms [11].

### 4.4. Lenalidomide

Early treatment for patients with high-risk SMM delayed disease progression and increased OS. Mateos et al. evaluated treatment in patients with high-risk SMM. High-risk MM was defined as BMPC ≥ 10% and a monoclonal component (IgG ≥ 3 g/dL, IgA ≥ 2 g/dL, or Bence Jones > 1 g/24 h) or only one of the two criteria described above, plus aPCs/BMPC ≥ 95%, with decrease of one or two uninvolved immunoglobulins ≥25%, without CRAB. They randomly assigned 119 patients with high-risk SMM to lenalidomide-dexamethasone treatment or observation. Lenalidomide-dexamethasone treatment significantly delayed disease progression and improved OS. The three-year PFS for patients in the treatment arm was 77% versus 30% for those in the observation arm (*P* < 0.001). 94% of the patients in the treatment arm versus 80% in the observation arm were alive at three years (*P* = 0.03) [12].

### 4.5. Other Agents

Some phase II trials are ongoing to determine whether the use of agents such as siltuximab (anti-IL-6 mAb), elotuzumab, MLN9708 (ixazomib), or BHQ880 (anti-DKK1 neutralizing Ab) is active in high-risk SMM [36]. The results will help provide more evidence and effective strategy to early treatment for patients with high-risk SMM.

## 5. Summary and Future

Currently, treatment is not recommended for SMM based on data derived from several small randomized controlled trials. However, the recent Mateos et al. trial showed improved OS with lenalidomide and dexamethasone treatment among patients with high-risk SMM. This suggested that high-risk SMM should be targeted for early intervention. However, the Mateos et al. trial had a small sample size. Much larger trials that select patients with high-risk SMM are needed to provide more evidence. The Mateos et al. trial used a combination regimen; therefore, whether the benefit was due to lenalidomide is unclear. More data were needed to isolate the effect of lenalidomide, and more trials were needed to evaluate the effect of other novel drugs which are not as expensive as lenalidomide on high-risk SMM. In addition, the criteria for high-risk SMM should be established. Mateos et al. used two separate criteria to identify high-risk SMM, one defined by Kyle et al. and another by Pèrez-Persona et al. This method was validated to identify patients who progress to active MM within almost 2 years after diagnosis. However, such criteria limit the general applicability of this approach because flow cytometry was required. Currently, a number of parameters have been described to predict risk of progression to symptomatic MM, prompting the following questions: (1) what probability of 2-year progression to symptomatic MM will be defined as high risk? (2) Which predictors should be used alone or in combination to identify high-risk SMM? Currently, there is no consensus, but it is generally accepted that patients with BMPC ≥ 60%, FLC ratio ≥ 100, or abnormal MRI (>1 focal lesion) are at high risk and need to be treated immediately. After reviewing all of the data, patients with BMPC ≥ 60%, FLC ratio ≥ 100, or abnormal MRI (>1 focal lesion) have ≥70% probability of progression to active MM (Table 3). If indeed there is a 70% probability of progression, we recommend that high PBPC also be considered as a high-risk factor.

## Figures and Tables

**Table 1:** Studies predicting risk of progression of SMM to symptomatic MM.

Author [year]	Included number and criteria	Risk factors	Outcome
Kyle et al. [2007] [15]	276 IMWG	Group 1 (BMPC ≥ 10% and M protein ≥ 30 g/L); group 2 (BMPC ≥ 10% and M protein < 30 g/L); group 3 (BMPC < 10% and M protein ≥ 30 g/L)	TTP: group 1: 2 y; group 2: 8 y; group 3: 19 y

Kastritis et al. [2013] [16]	96 IMWG	Risk factor 1: M protein ≥ 30 g/L; risk factor 2: extensive BM infiltration ≥ 60%; risk factor 3: FLC ratio ≥ 100; risk factor 4: abnormal MRI	TTP: with risk factor 1 was 2 y (versus 8 y without risk factor 1); with risk factor 2 was 15 m (versus 90 m without risk factor 2); with risk factor 3 was 18 m (versus 73 m without risk factor 3); with risk factor 4 was 15 m (versus not reached without risk factor 4).

Rajkumar et al. [2011] [17]	655 IMWG	BMPC ≥ 60%	2-y progression rate: 95%; TTP: 7 m

Pérez-Persona et al. [2007] [19]	93 IMWG	Group 1: neither aPCs/BMPC ≥ 95% nor immunoparesis; group 2: aPCs/BMPC ≥ 95% or immunoparesis; group 3: aPCs/BMPC ≥ 95% and immunoparesis	5-y progression rate: group 1: 4%; group 2: 46%; group 3: 72%

Pérez-Persona et al. [2010] [20]	61 IMWG	aPCs/BMPC ≥ 95%	3-y progression rate: for evolving SMM with aPCs/BMPC ≥ 95% was 46% (versus 8% with aPCs/BMPC < 95%); for nonevolving SMM with aPCs/BMPC ≥ 95% was 15% (versus no progressions with aPCs/BMPC < 95%)

Larsen et al. [2013] [21]	586 IMWG	FLC ratio ≥ 100	TTP: FLC ratio ≥ 100: 15 m; FLC ratio < 100: 55 m

Dispenzieri et al. [2008] [22]	273 IMWG	BMPC ≥ 10%; M protein ≥ 30 g/L; FLC ratio ≥ 8; low-risk: 1 risk factor; intermediate-risk: 2 risk factor; high-risk: 3 risk factor	5-y progression rate: low-risk: 25%; intermediate-risk: 51%; high-risk: 76%

Witzig et al. [1994] [23]	57 BMPC > 10% without CRAB	Abnormal PBPC (an increase in number or proliferative rate of PBPC)	TTP: with abnormal PBPC was 0.75 y (versus 2.5 y without abnormal PBPC)

Bianchi et al. [2013] [24]	91 IMWG	High PBPC (absolute PBPC > 5000 × 10^6^/L and/or >5% cytoplasmic Ig positive PC per 100 PBMC)	2-y progression rate: with high PBPC was 71% (versus 25% without high PBPC); 3-y progression rate: with high PBPC was 86% (versus 34% without high PBPC)

Rosiñol et al. [2003] [25]	53 BMPC > 10%, M-protein > 30 g/L or light chain > 1 g, hemoglobin > 100 g/L, without CRAB	Evolving SMM (a progressive increase in M protein, a previously recognized MGUS and a significant higher proportion of IgA type)	TTP: with evolving SMM was 1.3 y (versus 3.9 y with nonevolving SMM)

Moulopoulos et al. [1995] [26]	38 BMPC > 10%, hemoglobin > 105 g/L, normocalcemia, M protein < 45 g/L, and no lytic bone lesion	Abnormal MRI	TTP: with abnormal MRI was 16 m (versus 43 m with normal MRI)

Hillengass et al. [2010] [27]	149 IMWG	Focal lesions > 1	2-y progression rate: 0 or 1 focal lesion: 20%; >1 focal lesion: 70%

Neben et al. [2013] [28]	248 IMWG	del (17p13), t(4;14), +1q21 and hyperdiploidy	TTP: with del (17p13) was 2.04 y (versus 5.62 y without del (17p13)); with t(4;14) was 2.91 y (versus 5.71 y without t(4;14)). 3-y progression rate: with +1q21 was 43% (versus 27% without +1q21); with hyperdiploidy was 35% (versus 29% without hyperdiploidy)

Rajkumar et al. [2013] [29]	351 IMWG	Low-risk: no detectable abnormalities; Standard-risk: t(11;14), MAF translocations, other/unknown IgH translocations, or monosomy 13/del (13q); Intermediate-risk: trisomies alone; High-risk: t(4;14)	TTP: High-risk: 28 m; Intermediate-risk: 34 m; Standard-risk: 55 m; Low-risk: not reached

PC: plasma cells; PBPC: peripheral blood plasma cells; PBMC: peripheral blood mononuclear cells; Ig: immunoglobulin; MRI: magnetic resonance imaging; FLC: serum free light chain; BMPC: bone marrow plasma cells; aPCs/BMPC: aberrant plasma cells within the bone marrow plasma cells; TTP: median time to progression; CRAB: hypercalcemia, renal failure, anemia, and bone lesions; IMWG: International Myeloma Working Group; m: month; y: year.

**Table 2:** Clinical trials for patients with SMM.

Author [year]	Trial design	Therapy	Number and type of patients	Outcome
Hjorth et al. [1993] [7]	RCT	Initial versus deferred MP	50 stage I MM (DSS)	No difference in RR, response duration, or OS

Riccardi et al. [2000 and 1994] [5, 6]	RCT	Initial versus deferred MP	145 stage I MM (DSS)	No difference in RR or OS

Rajkumar et al. [2003] [8]	Single-arm phase 2	Thalidomide	16 SMM or IMM	11 of 16 patients responded to therapy

Martín et al. [2002] [32]	Single-arm pilot	Pamidronate	12 SMM or IMM	Reduces bone turnover but has no antitumour effect

Weber et al. [2003] [9]	Single-arm phase 2	Thalidomide	28 SMM	RR was 36% and median time to remission was 4.2 m

Musto et al. [2008] [35]	RCT	Zoledronic acid versus observation	163 SMM	No difference in PFS and TTP; reduce skeletal-related events

Barlogie et al. [2008] [10]	Phase 2	Thalidomide with monthly pamidronate	76 SMM	4-y OS and PFS were 91% and 60%, respectively

D'Arena et al. [2011] [33]	RCT	Pamidronate versus observation	177 SMM	No difference in PFS, TTP, or OS; reduce skeletal events

Witzig et al. [2013] [11]	RCT	Thalidomide plus zoledronic acid versus zoledronic acid	68 SMM	Significant difference in TTP and PFS; no difference in OS

Mateos et al. [2013] [12]	RCT	Lenalidomide plus dexamethasone versus observation	119 high-risk SMM	TTP: treatment: not reached; observation: 21 m; 3-y OS: treatment: 94%; observation: 80%

MP: melphalan and prednisone; DSS: Durie and Salmon stage; RR: response rate; OS: overall survival; PFS: progression-free survival; RCT: randomized controlled trial; IMM: indolent multiple myeloma; m: month; y: year.

**Table 3:** Risk factors predicting high-risk SMM.

Risk factors	Patients with risk factors accounting for the population of SMM	Probability of 2-year progression to symptomatic MM
Bone marrow plasma cells ≥ 60%	2–10%	90%
Serum-free light-chain ratio ≥ 100	15%	80%
Abnormal magnetic resonance imaging (>1 focal lesion)	15%	70%
High peripheral blood plasma cells^a^	15%	70%

^a^Defined as absolute peripheral blood plasma cells > 5000 × 10^6^/L and/or >5% cytoplasmic immunoglobulin (Ig) positive plasma cells per 100 peripheral blood mononuclear cells.
